# The Soundscape of Neonatal Intensive Care: A Mixed-Methods Study of the Parents’ Experience

**DOI:** 10.3390/children8080644

**Published:** 2021-07-27

**Authors:** Maria Chifa, Tamar Hadar, Nina Politimou, Gemma Reynolds, Fabia Franco

**Affiliations:** 1 Psychology Department, Faculty of Science and Technology, Middlesex University, London NW4 4BT, UK; MC1785@live.mdx.ac.uk (M.C.); G.Reynolds@mdx.ac.uk (G.R.); 2 Division of Expressive Therapies, Graduate School of Arts & Social Sciences, Lesley University, Cambridge, MA 02138, USA; Thadar@lesley.edu; 3 Institute of Education, University College London, London WC1H 0AA, UK; n.politimou@ucl.ac.uk

**Keywords:** prematurity, sound in NICUs, premature infant outcomes, parent perinatal stress and PTSD

## Abstract

Parents who have infants hospitalised in neonatal intensive care units (NICUs) experience high levels of stress, including post-traumatic stress disorder (PTSD) symptoms. However, whether sounds contribute to parents’ stress remains largely unknown. Critically, researchers lack a comprehensive instrument to investigate the relationship between sounds in NICUs and parental stress. To address this gap, this report presents the “Soundscape of NICU Questionnaire” (SON-Q), which was developed specifically to capture parents’ perceptions and beliefs about the impact that sound had on them and their infants, from pre-birth throughout the NICU stay and in the first postdischarge period. Parents of children born preterm (*n* = 386) completed the SON-Q and the Perinatal PTSD Questionnaire (PPQ). Principal Component Analysis identifying underlying dimensions comprising the parental experience of the NICU soundscape was followed by an exploration of the relationships between subscales of the SON-Q and the PPQ. Moderation analysis was carried out to further elucidate relationships between variables. Finally, thematic analysis was employed to analyse one memory of sounds in NICU open question. The results highlight systematic associations between aspects of the NICU soundscape and parental stress/trauma. The findings underscore the importance of developing specific studies in this area and devising interventions to best support parents’ mental health, which could in turn support infants’ developmental outcomes.

## 1. Introduction

This paper aims to focus on the parental experiences associated with the specific soundscape of neonatal intensive care units (henceforth NICUs). The literature has provided a wealth of information about various developmental aspects associated with infants’ early sonic experiences, including those in NICUs. In order to provide a rich picture of the parent–infant dyad’s experience with the NICU soundscape, we will first review relevant studies concerning the infants’ early development, but we will then focus specifically on the parents’ experience with sounds in NICUs. 

It is well established that newborns prefer the sound of a human voice (particularly speaking or singing) over and above any other auditory stimulus, likely to be for the adaptive function of orienting towards conspecifics [1]. As part of well-known prenatal auditory learning [2,3,4,5,6,7], infants recognise their mother’s voice [8] and their orientation to it is part of neonatal paediatric-behavioural assessments [9]. Early parent–baby vocal exchanges are important not only for establishing the basis of vocal communication, but also for bonding and attachment [10]. Therefore, vocalisation and sound are crucial aspects of early development and parenting [11]. This raises the question as to what happens when the typical natural path of auditory, vocal and bonding development is disturbed by premature birth and an often-prolonged period in neonatal intensive care. 

Every year, approximately 15 million infants are born before term worldwide [12] with around 60,000 in the UK [13]. According to Bliss [14], each year in the UK, 1 in 7 infants are cared for in NICUs, for various reasons such as meconium aspiration syndrome (MAS), asphyxia (lack of oxygen at birth), jaundice, hypoglycaemia (low blood sugar), infection or breathing problems and most commonly, prematurity [13]. An infant is considered premature, if s/he is born earlier than 37-week gestation (extremely preterm: <28 weeks; very preterm: 28–32 weeks; and moderately to late preterm: 32–37 weeks). The average length of the stay in NICUs for infants born under and up to 27-week gestation is 92 days, compared to 4 days of hospitalisation for infants born at term (over 37-week gestation) [15]. Risks of delays or deficits in cognitive and language development are more prevalent in preterm than term births [15,16,17,18,19]. A small loss of hearing or difficulty with attention can have a profound effect on learning, communication and emotional bonding [16,17,18,19,20,21,22]. The aetiology of these weaknesses is likely to be multifactorial [23], with risk factors being related to preterm birth and the iatrogenic effects of neonatal intensive care [23,24].

When considering the soundscape to which an infant is exposed, in the womb, a foetus is exposed to the sounds of the maternal body physiology and environmental sounds (including voices and music), transmitted through the amniotic fluid, conducted through bones and filtered by maternal body muscles, fat and tissue [25]. Conversely, infants in NICUs are exposed to a soundscape including hospital staff and their workings and, in particular, sounds from a variety of machines and medical equipment, which are intended to save infants’ lives and help them thrive (see Supplementary Materials: Table S1 for a list of equipment associated with sounds in NICUs). These sounds are transmitted through air coming from various directions, depending on the location of the source. Many nurseries exceed the sound levels of 45 dB (65 dB at peaks) recommended by the American Academy of Paediatrics (AAP) [26,27,28,29]. 

Excessive sounds from NICUs can negatively impact a preterm infant, affecting her/his development and the discharge period, by producing physiological changes in blood pressure, heart rate, breathing rate and oxygenation. This causes autonomic instability [30] by being distracted during feedings [31]; having adverse effects on the neuroendocrine and immune system [32], impacting sensory functioning (e.g., tactile abilities) [33] and interfering with sleep, which plays an important role in consolidating auditory memories in the prenatal period [34]. Considering that auditory learning begins from around 28-week gestation [35], instead of learning about the mother’s voice, speech, patterns of language and music, accompanied by tactile and vestibular stimulation associated with her breathing and movements as it happens in the womb [25], in NICUs, the voices are masked by electronic, non-biological sounds. These may occur 24 h a day, making it difficult to distinguish foreground from background sounds at 60 db or to filter out and process noxious stimuli [36,37,38]. In this environment, the preterm infant is being deprived of the exposure to the human voice and speech, which is associated with the neurobiology of language development [39,40,41,42,43,44,45,46,47,48,49]. 

Despite the literature showing the negative impact that high levels of NICU noise have on the development of the preterm infant [50,51], there is a scarcity of research on the reduction of NICU sounds. Based on the Cochrane Central Register of Controlled Trials, Almadhoob and Ohlsson [52] selected studies that involved sound reduction for preterm infants and found only one quality study investigating the impact of silicone earplugs compared to that of a control group without earplugs, in infants of 34-week gestation. The results demonstrated a significant difference in developmental outcomes favouring the use of earplugs at 18 to 22 months. Additionally, private rooms on neonatal units were recommended to avoid excessive noise, although there is a risk that infants could be deprived of sensory stimulation if parental involvement is minimal [53].

In order to support an optimal environment for the auditory experience of infants in NICUs [54,55], it has been suggested that parents provide the most appropriate sound environment in skin-to-skin care, which also provides an opportunity to hear voice (talking, humming and singing to their baby) and body sounds [56]. Music has also been found to have beneficial effects on premature infants’ development [57,58,59,60], notably maternal humming/singing [21]. Some parents consider introducing recordings of instrumental music to their infants while in NICUs, which have been accompanied by a note of caution as, differently from the human voice, they do not provide any form of reciprocation, have no specific links to the uterine sensory and might possibly become just another noise [39,61]. Music therapy, defined as a live-performed musical intervention, which may facilitate the active involvement of parents [62], has been found to positively impact the vital bio-physiological functions [63] and support stress reduction and faster hospital discharge [64]. In sum, the literature clearly indicates that infants’ development is negatively affected by two major aspects of the NICU soundscape: the NICU noisy environment and the lack of exposure to parental voice, with some forms of musical interaction potentially playing a role in mitigating these negative effects. 

Besides infants, premature birth can also negatively impact parents. Instead of holding their baby, touching, talking and taking her/him home, the baby is rushed to the NICU, exposing the parents to the overwhelming NICU soundscape which, according to Segal, is “a combination of control tower, server room and busy canteen” [65]. This interruption in the typical transition to parenthood requires parents’ adaptation and acceptance of the real infant, compared to their thoughts about the expected infant, making it harder to be identified as parents [66,67,68]. Additionally, parents may experience high levels of stress and helplessness [69], the inability to always be part of their infant’s care [70], the need to leave their infant in the hospital feeling like “an amputation, over and over” [65] and the worry about their infant’s developmental outcomes both while in NICU and at home [71,72]. All these aspects may contribute to the emergence of symptoms of post-traumatic stress disorder (PTSD) [73,74], which can affect the quality of the child–parent relationship and parenting skills [75]. 

Compared to the vast evidence supporting the impact that the NICU soundscape has on infants, the literature is far more limited with regards to the parents’ experience and whether they are negatively affected by it. There is evidence suggesting that excessive noise may impair hearing and trigger various diseases (e.g., cardiovascular disease) [76,77,78]. Perhaps, a more compelling finding is that parents experience high levels of stress as soon as they arrive at an NICU with their infants. For instance, Alkozei et al. [79] reported that 52% of mothers experienced high levels of stress at NICUs and 38% presented significant depressive symptoms. The study found that stress is not associated with demographic factors, pregnancy factors or mental health. However, NICU sounds and sights were mentioned as factors that contributed to the mothers’ level of stress, together with traveling long distances and marital status [79]. Feeley et al. [80] used a questionnaire to compare Canadian mothers of preterm infants in an open ward to those in a separate room. They explored the impact on symptoms of depression, readiness for discharge, sleep disturbance as well as the perception of staff–parent support. The two groups did not differ significantly on symptoms of depression or sleep disturbance, but the results found lower stress and higher readiness for discharge in mothers whose babies were in a separate room. Alternatively, mothers in an open ward reported higher sound stress and more restrictions in their parental role. However, infants’ conditions were not taken into consideration when forming the groups, which may have created uneven distributions of types and levels of preoccupation in the mothers. 

There is a large body of evidence showing that NICU parents are widely affected by their experience with prematurity. Suttora et al. [81] surveyed 87 mothers of premature infants and 156 mothers of full-term infants, with their children being from 1 to 36 months, to investigate the role of PTSD symptoms linked to childbirth in the development of parenting stress. The results showed that the mothers of full-term children reported fewer PTSD symptoms than the mothers of premature children. When applying the clinical threshold of the questionnaire used (Perinatal PTSD Questionnaire (PPQ)) [1], 55% of mothers of premature children and only 16% of mothers of full-term children, showed perinatal PTSD. Moreover, O’Donovan and Nixon [82] interviewed seven mothers and six fathers to explore parents’ experiences in the context of premature birth. They found that parents considered premature birth as traumatic, leading to overprotective parenting styles and hypervigilance. Fathers of preterm infants are underrepresented in the literature, and there are suggestions that they may respond differently to mothers regarding trauma such as lower anxiety levels with their difficulties being more rarely expressed [83]. However, similarly to mothers, Koliouli et al. [84] showed that fathers of preterm infants experience more symptoms of PTSD than their peers of full-term infants. Additionally, fathers might feel excluded from their babies’ care and discharge plans, which triggers a lack of confidence and increased stress in caring for their preterm babies [85]. Nonetheless, a significant percentage of both mothers and fathers present PTSD symptoms even beyond one year after their infant’s birth [86]. 

Parental trauma and PTSD symptomatology are important as infants of parents with PTSD are at higher risk of cognitive delays as well as behavioural and attachment issues [86,87]. Considering the negative associations between parental stress in NICUs and beyond the child development, and some sparse suggestions that the NICU soundscape may have an aversive impact not only on infants, but also on parents, the present study aims to specifically investigate the parental sonic experience of NICUs. Using a mixed-method design, a novel survey was developed in order to explore parents’ perception of the NICU soundscape and how it affects their ways of communicating with their infants. Four areas were studied: (1) the pre-birth soundscape and initial NICU environment; (2) parents’ observation of infants’ responses to NICU sounds and ways of communicating with their infants in NICUs; (3) the first postdischarge period at home; and (4) general impressions about the NICU environment. The first quantitative part of this paper will study the association between these areas of the sound experience in the perinatal context of premature infants and demographic variables and perinatal stress measured with the PPQ separately [88]). In order to triangulate aspects of the quantitative results with observations from parents, the second part of the paper will report qualitative analyses exploring some insights into the parents’ memories of sounds in NICUs coming from one open question in the survey.

## 2. Method

### 2.1. Participants

English-speaking participants (including non-native, fluent and native speakers) from English-speaking and other countries responded via social media such as Facebook, Instagram and WhatsApp, as well as via the researchers’ personal and professional networks (e.g., BLISS Baby Charity). Inclusion criteria were that participants had spent some time in NICUs with their infants and that their children were less than four years of age, to capture a fresh memory of the experience. In order to avoid the awakening of painful memories, parents who had lost an infant were excluded from taking part in this study. For the qualitative data, all the responses were included, regardless of the age of the child at the time of completion. A total of 1264 individuals took part initially. However, the majority of these respondents were eliminated for the following reasons: 189 participants did not qualify for the study (e.g., they did not spend any length of time in NICUs); 25 participants completed the survey, agreed consent initially but did not click “consent and submit” at the end of the survey; 15 participants left just before completing the demographic section; and 624 participants (49.4%) did not complete the survey having left it at various stages. From the remaining 411 participants that completed the survey, 25 participants were eliminated from the quantitative analyses because of having children outside the range reported above. A total of 386 participants (30.5%) were therefore included in the analyses with complete responses. Respondents were mainly female (97%). The mean age of the participants was 31.78 years (SD = 5.79). Detailed information about the sample is reported in Table 1, Table 2 and Table 3. 

### 2.2. Materials

(i) The sound in the NICU questionnaire—Soundscape of NICU Questionnaire (henceforth SON-Q)—was developed through the consultation with parents and relevant professionals. The questionnaire was designed to cover the following areas: (1) About your baby (demographics about infants, collected at the start of the questionnaire); (2) You and sound: Going to an NICU; (3) Your baby and sound in the NICU; (4) At home after being in the NICU; (5) About the NICU in general; and about you (demographics about parents, collected at the end of the questionnaire). Overall, the SON-Q included 32 questions articulated in 204 items evaluated on a 5-point Likert scale. Given that the SON-Q was long, due to its exploratory nature, we deemed it important to include some negatively worded items (e.g., “I did not notice vocalisations”) to introduce some “checks” of respondents’ sincerity and avoid acquiescence bias, thus increasing reliability [89]. Section (4) also presented one open question (optional), in which parents could share a memory they had from their experience in NICUs, which was associated with sound.
(ii) The PPQ was used to investigate the presence of PTSD symptoms in the parents. The PPQ comprises of 14 items scored on a five-point scale, ranging from 0 = not at all to 4 = often, more than a month (e.g., Did you have bad dreams of giving birth or of your baby’s hospital stay?). Higher scores are indicative of more severe PTSD symptomatology. There are also three subscales measured: intrusion symptoms, avoidance symptoms and hyperarousal symptoms.

### 2.3. Procedure

The SON-Q was constructed and distributed using the Qualtrics survey tool [90]. The parents were invited to complete the survey by clicking an online link, which was posted on social media and parent networks. Following the information section, the participants were first required to give their informed consent and then complete the SON-Q followed by the PPQ, ending with the demographic sections. The completion of the survey took no more than 60 min, depending on the amount of details that the respondents reported in the open question. However, the participants had the opportunity to take short breaks during the completion of the survey, with their answers being saved. The respondents had to click the “submit and agree to share anonymized data for publication” button at the end of the survey in order to be included as participants. The respondents did not receive any compensation for completing the survey. 

### 2.4. Data Analysis

Quantitative analysis: Data were analysed using IBM SPSS v25. First, principal component analysis (PCA) was employed for the four parts of the SON-Q in order to identify relevant dimensions underlying the data and possibly reduce the number of contributing items. Six factors extraction was implemented. The criteria used for factor extraction included Kaiser’s criterion (only factors with eigenvalues of >1 were retained) and visual inspection of the scree plot [91]. 

Next, Pearson’s correlations were used to investigate associations of interest, e.g., correlations between the different parts of the SON-Q and the PPQ scores, with alpha set at *p* ≤ 0.05. 

Based on the correlations, a linear regression model was built with backward elimination, to determine the variables that would be more explanatory with respect to perinatal stress. Finally, moderation analyses were employed to further elucidate relationships between variables. 

Qualitative analysis (SON-Q open question): A thematic analysis of the parent’s narrative data, transcribed verbatim from the SON-Q open question, was conducted, which offered the opportunity to triangulate aspects of the quantitative results with qualitative analyses. Guided by the steps of thematic analysis [92], the first author initially engaged in reading through the data several times and making notes. In the second phase, the data were organised into tentative codes, focusing on the most prominent memories parents mentioned in relation to their experiences with sound in the NICU. After this, through team discussion, the codes were further organised into themes, and finally, these themes were defined and named. 

### 2.5. Ethics

The research proposal was approved by the Psychology Research Ethics Committee of Middlesex University (#8279) as conforming to the ethical principles of the British Psychological Society and the WMA Helsinki Declaration. The participants received information about the study before participation, explicitly consented to take part in the survey and were provided with full debriefing, which included information about sources of support for participants, if needed. All data were collected anonymously. 

## 3. Results

The results are presented in two sections: (i) quantitative analyses of the SON-Q; (ii) qualitative data analysis (open question).

### 3.1. SON-Q Quantitative Analyses Results

With the aim of identifying meaningful and cohesive dimensions of the sound in the NICU experience and to reduce the initial number of the items of the SON-Q for the ease of subsequent analyses, a PCA with promax rotation on the SON-Q items was conducted. The Kaiser-Meyer-Olkin (KMO) measure verified the sampling adequacy for the analysis (KMO = 0.677; *p* < 0.001). An initial analysis was run to obtain eigenvalues for each factor in the data. The scree plot was ambiguous and showed inflexions that would justify retaining at least 10 factors. Six factors were extracted based on eigenvalus of >1 (factor loadings after rotation can be found in Table S2 of Supplementary Materials). Items loaded negatively and/or on more than one factor were eliminated.

All factors presented high or moderate reliability. The items that cluster on the same factor suggest that F1 represents “A challenging NICU soundscape” (Cronbach’s *α* = 0.867); F2 represents “Infants’ reactions towards noises and voices in NICU” (Cronbach’s *α* = 0.900); F3 represents “Singing and using recordings for infants in NICU” (Cronbach’s *α* = 0.913); F4 represents “Interacting and bonding with infants in NICU” (Cronbach’s *α* = 0.754); F5 represents “Parenting confidence (parental attention to infants’ sounds)” (Cronbach’s *α* = 0.810); F6 represents “Perception of the home environment” (Cronbach’s *α* = 0.815). Each of the factors was explored further in combination with the qualitative data (final questionnaire items can be found in Questionnaire S1 of Supplementary Materials). Correlational analyses were then conducted between birth weight and parental age and all factors of the sound in the NICU questionnaire. As can be seen in Table 4, the ages of the parents were significantly associated with the lower intensity of infant reactions towards voices and sounds in the NICU. Parental age was also negatively associated with higher scores in “Parenting confidence”. Interestingly, the lower the birth weight, the more attention the parents paid to infant reactions and the more the singing and interacting–bonding was reported. Furthermore, the lower the birth weight, the higher the engagement of the parents by using singing and recordings and the higher the behaviours towards bonding with the infants. Finally, there were significant associations among most of the factors of the SON-Q. 

Next, correlational analyses were conducted between birth weight and parental age with the PPQ-Total Score and PPQ-Intrusiveness, PPQ-Arousal and PPQ-Avoidance scales separately. As can be seen in Table 5, no significant associations emerged with infant birth weight, but the PPQ-Total Score and subscales showed significant negative associations with parental age. 

Finally, correlational analyses between all factors of the SON-Q and the PPQ-Total Score and subscales are shown in Table 6. The more challenging the sound environment in the NICU perceived, the more perinatal stress, intrusiveness, avoidance and arousal the parents experienced. Similarly, the higher their attention and tuning to their infants’ reactions, the higher the perinatal stress. Lastly, a perception of the home environment was negatively associated with all PPQ subscales. 

To explore the independent contributions of the different dimensions of the SON-Q in perinatal stress, a linear regression model was built with backward elimination, using perinatal stress (PPQ-Total Score) as a dependent variable and the “NICU soundscape”, “Infant Reactions”, “Singing-Recordings”, “Interacting–Bonding with Baby”, “Parenting Confidence”, “Home environment” and parental age as independent variables. This was carried out in order to determine whether which model would be more explanatory and parsimonious in explaining variance in perinatal stress, after progressively removing different predictors based on the F criterion (see process and all models in Table S3 of Supplementary Materials). As can be seen in Table 7, in the final model, “NICU Soundscape”, “Home Environment”, “Interacting–Bonding with Baby” and Parent age significantly contributed to the variance in the PPQ-Total Score, suggesting that all these variables have a significant influence on perinatal stress. Interestingly, the sound in the NICU explained the biggest amount of variance in the PPQ-Total Score. No obvious patterns were observed, and residuals did not appear to deviate from a straight line in the model, therefore meeting the assumptions.

Finally, based on theoretical considerations, the relationship between NICU soundscape and perinatal stress was explored to identify whether it was modified by either singing and using recordings for infants in the NICU or interacting and bonding with infants in the NICU. A moderation analysis using PROCESS in SPSS was conducted. Two models were built, where the outcome variable was perinatal stress and the independent variable was NICU Soundscape. In the first model, the moderator evaluated was the Singing-Recording subscale, and the results showed that the interaction was not significant (*B* = −0.002; 95% C.I.: (−0.008, 0.004); *p* = n.s.). In the second model where the moderator variable was Interacting–Bonding with Baby in the NICU, and the interaction approached significance (*B* = −0.03; 95% C.I. (−0.07, 0); *p* = 0.06). The conditional effect of a “challenging NICU soundscape” on perinatal stress was weakened in the function of the values of interacting-bonding with the baby: at low values of Interacting-Bonding with Baby (−3.26), the conditional effect was strongest (*B* = 0.71; 95% C.I. (0.54, 0.88); *p* < 0.001); at middle values of Bonding with Baby (.00), the conditional effect was slightly lower (*B* = 0.60; 95% C.I.: (0.49, 0.70); *p* < 0.001); and at high values of Bonding with Baby (2.16), the effect was slightly weaker although still significant (*B* = 0.52; 95% C.I.: (0.40, 0.65); *p* < 0.001). With respect to the relationship between a “challenging NICU soundscape” and perinatal stress, it is interesting to note that the level of interacting and bonding with the infant was found to be a marginally significant moderator. That is, the parents that bonded more strongly with their infant were less affected by the challenging sound environment in terms of their stress levels. On the other hand, the parents whose bond with their infant was not perceived to be as strong were more negatively affected by the sound environment in the NICU.

### 3.2. Open Question Qualitative Analysis 

A total of *n* = 239 participants completed this question, and their answers disclosed their perceptions of NICU sounds. The findings reflect the thick and ever-changing texture of the NICU soundscape, highlighting feelings of stress and at times helplessness experienced by the parents. 

Four themes emerged (see Table 8) with each having two subthemes. The first theme captured possible influences of different mechanical sounds on the parents’ experience of the NICU (“Sounds from machines and various inanimate object: What’s a beep?”). The second theme illuminated parents’ reactions to the sounds originating from the medical staff and other families hospitalised in the NICU (“Sounds from human sources”). The third theme revealed the parents’ difficulty to connect to their own voice as parents taking care of their baby in the NICU (“Unheard sounds of parents—‘we couldn’t vocalize’!”). The fourth theme emphasised the importance of incorporating music into the NICU soundscape in order to support families in their stay at the hospital (“The sound of music as supporting parents and babies”). All themes and subthemes are presented separately below with verbatim extracts of participants’ responses. Each theme will be illustrated with full verbatim quotes from anonymised participants.

**Theme 1: Sounds of machines and various inanimate objects: What’s a “beep”?**

The majority of the participants mentioned memories of sounds originating from machines and various inanimate objects. This theme predominated the parents’ comments and formed the most rich and elaborated aspect of parents’ descriptions. The monitor alarm appeared most frequently amongst parents’ comments, in addition to other NICU machines such as the ventilator, opening–closing incubator doors and Bipap machines. Noteworthily, many of the parents’ comments revealed their awareness of sporadic and commonly unnoticeable sounds, such as the sound of a stapler, an opening of syringe packets, a trolley pushed around, the ring of doorbells, noisy shoes squeaking, speaker announcements, butterfly oscillator, the ringing of milk heaters, apron dispensers, on-hold music on the phone and the hums and the ticks of the clock while waiting. The parents mentioned their own responses to not only machine sounds, but also their babies’ sounds. This suggests that any noise in the NICU’s vicinity can trigger anxious responses based on personal circumstances and casual coincidences, due to the parents being susceptible to environmental signals.

“The soft beeping of the monitors is overwhelmingly etched in my memory” (P92).

“I’ll never forget the high pitch patterned beep if the UV light for jaundice and the alarm when heart rate went to low or oxygen went low” (P213).

“The sound of the doors opening; the sound of shoes on the highly buffed floors” (P100).

This theme was dominated by two subthemes: “Constant ‘beeping’ as a symbol of the parents’ ceaseless worrying”; and “The role of “beeps” as traumatic reminders”, as follows.

**Constant “beeping” as a symbol of the parents’ ceaseless worrying.**

The comments highlighted the perpetual “beeping” as reflecting the parents’ general experience of continual stress: constant sense of urgency and preoccupation over their babies’ health; continuous running; constant fear of losing their babies; an endless struggle to divide themselves between home and hospital; and an immeasurable desire to do more for their babies.

“The relentlessness of the sounds was overwhelming, but I think more than the sounds alone, was what they represented-danger; sickness; probable death. And it was that combination that I found torturous” (P176).

“During my son’s NICU stay his condition deteriorated rapidly. His belly was swollen and his oxygen saturation’s suddenly dropped whilst on bipap breathing support. The machines were getting louder and louder, doctors were running around taking turns to try and stabilise him. Another doctor was on the phone to a higher level NICU hospital for an emergency transfer. No one could tell me what was going on. Between the noisy machines, not being able to be near my son as there were four doctors working on him, the noise from doctors & nurses rushing. I was completely overwhelmed” (P34).

“I remember that every time the emergency alarm was on I felt that my world is breaking. I would let anything behind, run to my babies’ nursery and pray that they were well, and the alarm was not on because they needed help” (P1).

“Beeping. Incessant beeping. Of different tones and pitches but beeping. One time my baby’s oxygen monitor went off whilst I was feeding her but I couldn’t differentiate this as urgent, compared to all the other beeps that we going on” (P78).

The role of “beeps” as traumatic reminders. The comments reveal the complex and enduring effects of the alarm and portray sound as contributing to the parents’ overactivated nervous system, in many cases possibly resulting in PTSD. Parents’ comments emphasised the association of the sound with their babies’ clinical state, suggesting that they represented much more than acoustic signals. The comments revealed that once the parents transitioned with their baby to the home environment, the mechanical sounds became haunting, causing nightmares and bringing back vivid memories by associating everyday sounds to the ones of the NICU.

“Alarms going off is associated with my babies desats. I think I have PTSD according to my reaction to alarms” (P58).

“The Asystole and bradycardia and Apnea alarms haunt me” (P74).

“The low loud “boom boom” sound that the monitors made will stay with me forever. Whenever I look back at recordings of my son and I hear that noise in the background it makes me well up. It’s a haunting sound” (P117).

“Once a very sick baby crashed 3 times in 25 min in our ICU room. It was terrifying. I still have nightmares almost 2 yrs later. The crash alarm rings in my ears so loud. I’ll never forget it as long as I live” (P232).

“Regular beeping noise still create feelings of stress and anxiety for me” (P210).

“I just remember that for many years my son was terrified of sudden high pitches noises. He could go from floor straight into my arms at one leap if he heard something like a monitor sound. But could sleep through a Hoover next to his cot!” (P174).

Nevertheless, the findings emphasised that despite the “beeps” being distressing for parents, they also reassured them regarding their infant’s health. If given a choice, parents would more often prefer to hear a sound than not to, and feel relieved, against the uncertainty and fear that something adverse might be going on with their baby, portraying the “beep” sounds as a double-edged sword.

“Sounds are scary to start with but become reassuring” (P192).

“For a while after coming home, I’d have to put the radio on quietly in the bedroom. Bings and bongs were somewhat reassuring” (P149).

“My son used to suffer from desats where the monitor used to alarm if it happened which was quite frequent. When it was being discussed he could go home they turned off this monitor just a few days before discharge which I felt gave me anxiety because I got into a habit of relying on the noise to tell me when it was happening” (P124).

“When returning home in the evenings, I would constantly hear all the beeping from the machines in the NICU, on occasion I would wake up after hearing the urgent call response (although I was at home) prompting me to ring the NICU throughout the night. The noises from the machines were so reassuring yet so traumatic” (P138).

“The sounds of the monitors became familiar & strangely comforting, especially when I visited our daughter alone” (P118).

**Theme 2: Sounds from human sources.**

The parents’ comments suggested that the NICU soundscape did not include only desolate sounds from machines and objects, but also human sounds made by medical staff caring for the hospitalised infants as well as parents and families visiting the NICU.

**The sounds of medical staff.**

Parents’ comments showed that occasionally, they were disturbed by staff’s volume of speech and private conversations held in the vicinity of their baby’s bed.

“My daughter was very sensitive to sound. Even with signs reminding staff to talk softly they would talk very loudly, and her stats would drop” (P62).

“Staff talking too loud/laughing” (P11).

“Staff talking loudly and often about non-work-related matters” (P115).

**The sounds of other hospitalised families.**

The comments reveal the parents’ sensitivity to sounds originating from other families hospitalised in the NICU of adults as well as those of young siblings of NICU babies.

“Visitors of one of the babies were regularly very loud with children running and screaming which visibly affected my baby” (P132).

“Staff (rightly) weren’t in control of the volume of other parents, some at times were loudly derogatory about staff and I felt so torn about leaving my baby and felt so guilty knowing I was leaving her in such a toxic environment (thankfully this was the minority (but loudest!) of parents” (P 99).

“Other parents were loud” (P146).

**Theme 3: Unheard parents’ sounds: “we couldn’t vocalise!”.**

The findings revealed parents’ thoughts about their position in the NICU environment and their struggles and pain for not being able to practice parental skills. This was reflected in parents’ reports in two different ways: (1) on the one hand, the comments highlighted feelings of transparency and incapability; (2) on the other hand, the parents expressed feelings of helplessness relating to hearing other babies cry, while fearing their own baby might be subjected to similar moments of discomfort, when they were absent from the NICU.

**Parents’ feelings of transparency and incapability.**

Separated from their baby, by the alarming as well as the lively sounds of the NICU, seemed to induce a state of detachment on the parents’ side. The parents emphasised sensing a lack of their parental voice throughout their NICU stay. The parents reported that in trying to keep quiet, their thoughts and desires were suppressed, affecting their ability to communicate with their babies.

”I found it difficult to connect with my baby as it was too quiet, and I didn’t want to disturb the rest of the unit at times.” (P82).

“So quiet around us that we preferred not to talk rather than have everyone hear us. I was embarrassed and overwhelmed and just went into myself. I didn’t want to be there and I didn’t know what I was doing” (P88).

“I hadn’t held my baby and didn’t let myself feel anything for him so there was no questions of me singing or chatting to him in that situation. I had to wait until I was at home alone with him for that” (P88).

“… the fear of speaking too loud and/or making noise. Feeling like you had to be silent” (P166).

“My baby was taken off me by the NICU nurse and rubbed and manitoulated (sic) until the oxygen levels were restored. I’ve thought about that moment a lot that I could and should have done more but I didn’t because I didn’t know what this beeping was telling me” (P78).

**Parents’ perplexity towards other babies’ cry.**

In light of the absence of their parental voices, the comments illuminated the stress induced by hearing other babies in the NICU crying, which seemed to connect the parents even more to their sense of parental incompetence. The cry of other babies, in that sense, symbolised for the parents their inability to fulfil their parental roles and stand by their baby at all times, while being hospitalised in the NICU.

“The most heart-breaking noise for me was the crying of other babies when nobody was around to attend to them. I worried that when my daughter started to cry, she would be left in the same way. I really didn’t mind the alarms, but I hated hearing the other babies cry” (P204).

“…staff would often not have time meaning babies next to mine sat crying or their monitors went off and nobody appeared to look at them, it made me anxious did they ignore my baby when I wasn’t there?“ (P194).

“The crying of other people’s babies made me feel frustrated that we could not be at home enjoying each other’s company in our own bubble. As a first-time parent, you expect to enjoy a certain time of calm and to be used to your new arrival, the NICU attacks all your senses and everything you have prepared your baby for. The sounds are as unfamiliar to baby as they are to you” (P188).

**Theme 4: The sound of music as balancing the NICU’s cacophony.**

As opposed to the aforementioned themes, which focused mainly on the distressing aspect of the NICU soundscape, this theme reveals the power of music as contrasting and mitigating “harmful sounds” and as facilitating for both parents and their babies a more comforting and supporting experience.

**Music as a supporting agent for parents and babies in the NICU.**

The findings showed that music transformed the parents’ experience of the NICU in several ways: (1) music helped parents to feel at home and encouraged infant–parent bonding; (2) music induced a state of relaxation and comfort for babies and parents and, in some cases, supported them in processing their stressful experience of being hospitalised; (3) music in the NICU had an influence on infants’ development.

“I remember my baby’s night nurse would sing and hum as she did her rounds. It seems like when she hummed during feedings, the babies would take to the bottles better. When she hummed my baby would turn in her direction, or her heart rate would even out while she was being held and hummed to by her (much like she did being held by her parents). Sound is very important to babies, especially those in loud NICUs, in order to maintain a peaceful demeanor” (P68).

“Special care unit towards the end of our stay started to play music in the corridors between the nursery and it made all the difference to the parents as it made it feel less like a hospital setting” (P38).

“Whilst pregnant, I played piano everyday (I play to a high standard) and got heavily into a particular composer. His work will be forever associated with that time as after my daughter’s birth, I downloaded an album of his played work and played it to her throughout NICU and beyond. Her experience of NICU was very calm-she slept through it. Literally. I believe it was aided by as much kangaroo care as I could give, and music. Non-stop music that the nurses let me play to her” (P43).

“It seems like when she hummed during feedings, the babies would take to the bottles better” (P68).

**Music as a transitional object for families when entering their home environments.**

In addition, parents shared that music made the hospital–home transition smoother for them and for their infants. Being discharged from the NICU is a widely acknowledged stressful event, due to parents’ feeling wholly responsible for their infants’ health, without the hermetic protection of nurses and doctors, for the first time. The findings suggested that the same music parents chose to listen to in the NICU (and sometimes already when pregnant) served them as transitional objects when taking their baby home for the first time. This emphasises not only the important role of music in the NICU, but also its sustained impact over time and space.

“My husband and I slept with music on for two months after bringing our daughter home” (P197).

“There is a song by a band Athlete called ‘Wires’ written about their experience in NICU. Every time I hear it, I bawl my eyes out. Certain noises or in this instance songs bring back the vivid memories” (P154).

“I loved being able to sit in a rocking chair with my son with my worship music playing. He was the most active while I was pregnant listening to that music and the most relaxed in the NICU and once home” (P61).

## 4. Discussion

The results of this study present a systematic survey of the parent’s perception of the NICU soundscape, from the perspective of a large sample of parents of premature infants, from which perinatal stress measures were also collected. To the authors’ knowledge, this is the first attempt to capture specifically and in detail, from a parents’ perspective, a multifaceted representation of the parental experience of the NICU soundscape. For this purpose, the exploratory questionnaire, SON-Q, was developed to investigate parental experiences across the birth throughout their infant’s stay in an NICU and the initial postdischarge period at home. The quantitative results from the questionnaire were then triangulated with insights based on parents’ generated memories from one open question of the SON-Q. Overall, the quantitative and qualitative results have been highly convergent, showing that the NICU soundscape plays a central part in the parents’ most stressful, sometimes traumatic, experiences with their preterm infants while they were hospitalised. Although some studies suggest that there is no association between sounds in NICUs and parents’ stress [93], the findings from the present study are in line with those reported by Turan et al. [94], who found that parents in NICUs experience stress due to sound, among other factors such as sights, infants’ appearances or parental role alteration. 

The first evaluation of the SON-Q identified six factors with moderate to high reliability. In order to shed light on the parental concerns identified by these factors, the results of the quantitative analyses were complemented by the content analysis of the open question of the SON-Q, asking parents to share some of their more personal memories of the NICU soundscape. The themes that emerged from the qualitative analysis are consistent with and illuminate the interpretation of the dimensions of parental concern identified by the factors.

The first factor that emerged from the SON-Q, “A Challenging NICU soundscape”, captured parents’ beliefs with regards to the NICU soundscape and the impact that it had on them. Although the relationship between sound and stress is under-investigated, a review of the literature suggested that the NICU sound environment might trigger physiological and behavioural responses, which may negatively impact communication and job performance [95]. From the staff’s perspective, Santos et al. [96] outlined that the levels of noise from NICU were excessive, with an emphasis on noise from equipment. This factor suggests that parents also perceived the NICU soundscape as a negative environment, increasing their stress levels. They also emphasised that specific times of the day were noisier, making it challenging to communicate with their infants and concentrate on the care they needed to provide them, making various suggestions for improvements.

Moving the focus to the infants, ”Infants’ reactions towards noises and voices in NICU” portrays various reactions that parents observed in their babies, in particular towards noises from medical equipment and voices speaking or singing in NICUs. Due to the descriptive nature of the responses, it is difficult to assess whether the reactions were perceived as positive or negative ones. However, previous studies have shown that the exposure of premature infants to continuous loud noises, such as sounds from equipment, triggers negative physiological responses [97,98]. On the other hand, there is evidence that premature infants show positive reactions towards their mothers’ speaking and singing, which is consistent with the literature on infants’ sensitivity to the biologically meaningful and attractive sounds of their mother’s and other voices [21,99]. Suggestions from the qualitative data also highlight that inadequate volume of voices, such as staff talking or children visiting their siblings being particularly loud, also negatively impacted their infant’s reactions and would present challenges to understand speech and communication [38]. 

Interestingly, the third factor captured the specific aspect of musical communication, with “Singing and using recordings for infants in NICUs” representing parents’ beliefs about singing to their babies and using recordings in NICUs, such as keeping babies’ company, supporting their development, expressing thoughts and feelings and covering aversive noises in NICUs. These results are in line with findings showing benefits of singing for infants in NICUs [100,101], especially with live vocal contact that fosters multimodal co-regulation, intuitive parenting, unconscious mimicry, as well as reciprocal synchronization, with all vital aspects supporting attachment and bonding [102]. Whilst some studies emphasised that parents, staff and physicians would prefer recorded music for infants in NICUs [103], there is evidence suggesting that live music is far more beneficial for premature infants than recorded music [99,104]. Consistently, the parents revealed how singing to their babies encouraged bonding, but also highlighted that the NICU environment made them feel prone to suppress their wishes to communicate and sing to their babies, feel the need to be quiet and not to disturb other hospitalised infants and their parents, thus justifying somehow the use of recorded music.

The factor “Interacting and bonding with infants in NICUs” represents parents’ manifestations towards their babies while in NICUs, such as talking to them, holding hands on them and being able to discriminate a pain cry, which is in line with the literature emphasising the parents’ desire for physical contact [67,105]. Cogently, exposure to higher parent talk during infants’ hospitalisation is linked to increased infant vocalisation at discharge and better cognitive outcomes and language skills in toddlerhood [106]. However, the bonding process in NICUs is threatened by the aversive environment and the need for separation [107]. Despite the changes in the NICU towards a family-centred approach, with parents being increasingly involved in their babies’ care, intensive care still makes it difficult to experience parent–infant closeness [108]. This can cause parental depression and feelings of guilt, but also poorer developmental outcomes in infants [109]. Indeed, in the qualitative analyses, the parents revealed that they felt that bonding with their infants was affected by the NICU sound environment, suggesting that quiet interaction time with an improved soundscape, including music on the background, could promote this aspect.

Further developing the previous aspects, the factor “Parental confidence and attention to infants’ sounds” referred to not only various vocalisations produced by infants in the NICU, allowing the parents to identify them among other babies, but also various types of crying that parents were able to discriminate within their babies, such as pain and tiredness crying. Parent’s intuition about the importance of infants’ vocalisation is compatible with research investigating infants’ vocalisations in NICUs. While research is still relatively scarce, there is evidence suggesting that infants start to vocalise from as early as 32 weeks, which is an ability that increases over time [105], and that also includes non-cry vocalisations (‘protophones”) [110]. However, this ability could be affected by complications in premature birth and related interventions required (e.g., intubation) [111], which may in turn explain language delays in preterm infants [45,112]. 

Importantly, communicative signals and the amount of time spent in contact with an infant and the direct caregiving experience also modulated parental functioning. Despite these actions being constrained in the NICU, the parents from the present study still reported recognising their infant/s as well as their infants’ needs by the sounds they made, which may be due to “intuitive parenting” [113]. However, such parental perceptions might be influenced by various factors, such as culture or depression, but might be also related to their specific attention towards their infants’ clinical state, derived from their experience in NICUs, and the awareness that misunderstanding the signals can compromise the infant [114].

Finally, “Perception of the home environment” outlines a contrast in the parents’ beliefs about the home environment after the first postdischarge at home. On the one hand, they perceive the home environment as a negative one, which is consistent with robust research about the difficulties that premature infants’ parents experience after the first discharge at home. In particular, with difficulties in the transition to parenthood conflated with the transition from hospital to home, any unresolved issues in hospital continue at home (e.g., stress and anxiety [115], depression and grief [116], lack of confidence and self-efficacy in parenting [117] or a need for additional professional and social support [118]), which are some of the reasons why parents described their home environment as chaotic and discouraging in the present survey. On the other hand, discharge to home brings various benefits to parents, such as not having to do the tiring travel to the hospital [119], being able to bond with their infants without medical sounds and sights, as well as not being impacted by the dynamics of the NICU including other babies crying and variety of medical staff caring for the infants. The findings from this factor are in line with parents’ comments explored in the qualitative analyses, in which the majority of participants (73.6%) reported that their infants were discharged from the hospital without additional equipment, suggesting that their infants did not have severe health problems.

Besides the remarkable convergence between the quantitative and qualitative results, this study also revealed important relationships with demographic aspects and parental perinatal stress. For instance, the lower the birth weight, the more attention parents paid to infants’ reactions to sounds and the more singing and interaction–bonding reported. Although the literature indicates that premature birth can undermine the development of efficient parent–infant bonding [120,121], these findings highlight parents’ intuition or awareness of the vital role that interaction and bonding play in their infants’ development [122]. Moreover, reading the signals from the tiniest babies and trying to establish a connection with them might stimulate the parents to find a place for themselves in their infants’ life to be identified as parents [122,123,124]. In other words, even if their babies cannot respond, the parents still activate their parenting schemes.

Regarding the relationship between perinatal stress and the NICU soundscape, PPQ scores were higher in parents who perceived the NICU sound environment as aversive and challenging and who had infants with very-low birth weight, which is consistent with previous research suggesting that the NICU environment is overwhelming [125,126]. There was no overall significant relationship between PPQ scores and the birth weights of the infants, which is inconsistent with the literature suggesting that maternal PTSD is linked to low birth weight [127,128] but align with the suggestion that all parents who experience an NICU stay can potentially display PTSD symptoms, regardless of how preterm an infant was born [87,129]. However, the moderation analysis revealed that the PPQ scores in parents who had a very low-birth-weight baby were higher for younger parents than for older parents. This might be explained by the fact that apart from the baby being unwell, the younger parents might be experiencing various other difficult situations [130], such as lower levels of education [131], unplanned pregnancy [132], unstable material situation or unclear marital status [133].

Indeed, it has been shown that parental posttraumatic stress and trauma adversely affect parenting and bonding, thus posing a risk for children’s developmental outcomes [134,135]. Thus, it is interesting to note that the level of interacting and bonding with the infant was found to be a marginally significant moderator in the relationship between the NICU soundscape and perinatal stress. Specifically, the parents that bonded more strongly with their infant were slightly less affected by the challenging sound environment in terms of their stress levels. On the other hand, a trend was observed for parents whose bond with their infant was not perceived as strong to be more negatively affected by the sound environment in the NICU.

In sum, the novel findings form this study are substantially consistent with the relevant literature. However, a few limitations of this study might be considered. First of all, this is an exploratory correlational study; hence causal interpretations cannot be made. Only a randomised controlled trial comparing parental stress in an equivalent population exposed to different NICU soundscapes could establish a possible causal relationship between characteristics of the NICU soundscape and parents’ stress and trauma levels. Second, the numerosity of the sample was relatively small for the particular statistical analysis performed, i.e., PCA (recommended number of participants per item [91]. However, the other necessary conditions for PCA were consistent with the use of this form of analysis for the SON-Q data, which will be further elaborated to create a reduction/simplification of the items and develop a leaner version of the questionnaire to be used in future research. It is important to consider that the sample is per se substantial at *n* = ~400, since the proportion of parents who had an infant in an NICU represents a small percentage of infants [13], respectively, 1 in 7 babies is admitted to NICU each year in the UK [15] where the data were collected, suggesting that a high number of these parents wanted to express their views on their sonic experience in NICU. The sample was further limited by two potential biases: (i) gender bias—97.9% of the respondents were female, which is congruent with the literature available, suggesting that fathers of premature infants were underrepresented in our sample [85]; (ii) selection bias—more than 50% of respondents who engaged with the survey did not complete it. Although this is not uncommon in online surveys, it is possible that demographic variables (e.g., education) affected this decision, we were not able to compare demographic variables between respondents who did and did not complete the survey at the end of the survey due to the parent demographic section.

In spite of the acknowledged limitations above, the present study provides an unprecedented level of details in consideration of the importance of the soundscape in the early stages of the parental experience, particularly for those parents experiencing traumatic scenarios at the beginning of the lives of their infants. 

## 5. Conclusions 

This study was successful in developing a novel tool to investigate parents’ experience with the soundscape in NICUs. The study highlights associations between the soundscape in NICUs and parental stress. Additionally, it provides initial suggestions to stimulate further research, audience involvement and interdisciplinary collaborations aiming to improve parents’ mental health, as well as supporting early bonding and facilitating positive developmental outcomes for infants in need of intensive care. Some practical considerations and suggestions for future research are as follows:

(i) It is important to address parents’ needs in order to support their mental health. For instance, being delivered in a family-centred approach in combination with Kangaroo Care, music therapy in NICUs has been shown to be beneficial, helping to reduce parents’ anxiety and stress and improve their mood, restfulness and motivation [136]. Additionally, it improves breastfeeding [137], relaxation [138] and parent–infant bonding [100,139], which is supported by emerging parental identity [140].
(ii) Future research is important to extend this study cross-culturally and to investigate the sonic experience of premature infants and their parents during both their NICU stay and early postdischarge across a variety of cultural and social contexts [141,142,143,144]. The convergent quantitative/qualitative results demonstrated the importance of opening a conversation between parents who have experience in NICUs, medical and nursing staff, psychologists and engineers, to plan strategies for improving the sonic experience in NICUs. This is crucial for both infants and parents, encompassing support for parent–infant vocal interaction and the mitigation of noxious aspects of the NICU soundscape, especially those derived from medical machines.
(iii) In this respect, future research may involve partnerships with technology developers targeting the overall improvement of the NICU soundscape. The present study focuses on the subjective parental experience with the NICU soundscape, but two important points need to be kept in mind. Indeed, different brands of the same equipment may use more/less loud or unpleasantly pitched signals and hence may affect the NICU soundscape in different ways; similarly, the layout and space available in the wards may also increase/diffuse noxious sound effects. Last but not least, the noisy equipment in NICUs in many cases has a life-saving function, and hence, the relevant question is not about having or not having the equipment, but regarding implementing changes in which signals could be designed either based on current understandings of human emotional responses to sounds with different characteristics or based on the exploration of other sensorial modalities (e.g., vibration) or a combination of sensorial modalities allowing for different levels of sensitivity. Objective measures of specific acoustical parameters should be the basis for new standards and future interdisciplinary studies comparing outcomes in both infants (e.g., auditory development) and parents (e.g., perinatal stress affecting their parenting ability and coping) across environments.

## Figures and Tables

**Table 1:** Descriptive statistics of the sample (parents and infants).

	M	SD	Minimum	Maximum
Age of the respondents (years)	31.78	5.79	18	53
Gestational age at the birth of the infants (weeks)	30.79	4.27	22	42
Days spent in NICUs (N)	53.03	48.78	1	327
Infant birth weight (kg)	1.614	0.839	0.420	4.490
Age of child when the survey was completed (months)	17.96	11.86	1	46

**Table 2:** Infant sample characteristics.

Gender of the Child	Cause of the Birth	Categories of Weight	Categories of Prematurity	Status	Equipment Infants Were Discharged with
199 (51.6%) Female	90 (23.3%) Preterm rupture of membranes	111 (28.8%) Extremely low birth weight	113 (29.3%) Extremely preterm	329 (85.2%) Singleton	284 (73.6%) None
187 (48.8) Male	79 (20.5%) Preeclampsia	84 (21.8%) Very low birth weight	113 (34.5%) Very preterm	51 (13.2%) Twins	67 (18.4%) Oxygen Concentrator
	16 (4.1%) Elective preterm delivery	142 (36.8%) Low birth weight	113 (29.3%) Moderately to late preterm	1 (0.3%) Triplets	16 (4.1%) Feeding pump
	201 (52.2%) Other *	49 (12.7%) Normal birth weight	27 (7.0%) Full-term	5 (1.3%) Other	5 (1.3%) Saturation monitor
					3 (0.8%) Optiflow
					3 (0.8%) CPAP

* Other causes of birth mentioned by parents are placenta abruption, spontaneous labour, HELLP, incompetent cervix, gestational diabetes, absent diastolic flow, IUGR, chorioamnionitis, haemophilus influenzae, septicaemia, abnormal dopplers, uterine infection, obstetric cholestasis, uterine rupture and reduced movements of the foetus.

**Table 3:** Respondents’ demographic characteristics in order of prominence in the sample.

Gender	Ethnicity	Occupational Groups	Highest Level of Education Achieved	Years Spent in Education
378 (97.9%) Female	361 (93.5%) White	111 (28.8%) Intermediate managerial/ professional/ administrative	193 (50%) College or university	162 (42%) 14–18
7 (1.8%) Male	8 (2.1%) Asian/Asian British	101 (26%) Supervisory or clerical/junior managerial	94 (24.4%) Postgraduate (e.g., Master)	108 (28%) Over 18
1 (0.3%) Prefer not to say	6 (1.6%) Mixed	48 (12.4%) Skilled manual Worker	69 (17.9%) Higher/ secondary	59 (15%) 12–14
	3 (0.8%) Black British	36 (9.3%) Higher managerial/ administrative	26 (6.7%) Secondary up to 16 years of age	40 (10.4%) 9–12
	6 (1.6%) Other	24 (6.2%) Homemaker	3 (0.8%) Postgraduate (e.g., doctorate)	9 (2.3%) 6–9
	2 (0.5%) Prefer not to say	48 (17.3%) Other	1 (0.3%) Primary school	8 (2.1%) Less than 6

**Table 4:** Bivariate correlations between birth weight, parental age and all factors of the SON-Q.

	Birth Weight	NICU Soundscape	Infant Reactions	Singing-Recordings	Interacting-Bonding with Baby	Parenting Confid.	Home Environ.
Child Age		−0.04	0.01	0.00	−0.06	−0.09	−0.02
Parent Age	−0.06	−0.05	−0.15 **	−0.04	0.08	0.26 **	0.09
Birth Weight	-	−0.12 *	−0.16 **	−0.22 **	0.15 **	0.04	0.01
NICU Soundscape			0.31 **	0.08	0	−0.03	−0.04
Infant Reactions				0.26 **	0.10	0.31 **	−0.05
Singing-Recordings					0.31 **	0.29 **	0.16 **
Bonding with Baby						0.14 **	0.22 **
Parenting Confidence							0.10 *

* *p* < 0.05; ** *p* < 0.01. Note: Child Age = Child Age at the time of the questionnaire completed; Parent Age = Parental Age; NICU Sound = Challenging NICU soundscape factor; Infant Reactions = Infants’ reactions towards noises and voices in the NICU; Singing-Recordings = Singing and using recordings for infants in the NICU; Interacting–Bonding with Baby = Bonding with infants in the NICU; Parenting Confid. = Parenting confidence (parental attention to infants’ sounds); Home Environ. = Home Environment.

**Table 5:** Bivariate correlations between birth weight, parental age and all subscales of the Perinatal Post-Traumatic Stress Disorder Questionnaire (PPQ).

	Birth Weight	PPQ-Total Score	PPQ-Intrusiveness	PPQ-Avoidance	PPQ-Arousal
Child Age		0.01	0.03	0.04	−0.02
Parent Age	−0.05	−0.12 *	−0.12 *	−0.10 *	−0.10 *
Birth weight	-	0.05	−0.03	−0.05	−0.05
PPQ-Total Score		-	0.81 **	0.92 **	0.90 **
Intrusiveness			-	0.64 **	0.66 **
Avoidance				-	0.74 **

* p < 0.05; ** p < 0.01. Note: Child age = Child age at the time of questionnaire completion; Parent Age = Parental Age.

**Table 6:** Bivariate correlations between birth weight, parental age and all factors of the sound in the neonatal intensive care unit (NICU) questionnaire and all PPQ subscales.

	PPQ Total Score	PPQ-Intrusiveness	PPQ-Avoidance	PPQ-Arousal
NICU soundscape	0.46 **	0.42 **	0.43 **	0.39 **
Infant Reactions	0.12 *	0.19 **	0.05	0.13 *
Singing-Recordings	0.01	0.07	−0.04	0.04
Interacting-Bonding with Baby	0.03	0.06	−0.02	0.07
Parenting Confidence	−0.04	0.00	−0.05	−0.05
Home Environment	−0.28 **	−0.19 **	−0.30 **	−0.24 **

* *p* < 0.05; ** *p* < 0.01.

**Table 7:** Final regression model predicting the PPQ-Total Score after progressively removing “Singing-Recordings”, “Parenting Confidence” and “Infant Reactions”.

	*β*	*t*	*p*	*R* ^2^	*F*	*p*
Final model				0.30	42.45	<0.001
NICU Soundscape	0.45	10.60	<0.001			
Home Environment	−0.28	−6.54	<0.001			
Bonding with Baby	0.10	2.35	<0.05			
Parent Age	−0.08	−1.9	=0.05			

**Table 8:** Qualitative analysis of themes and subthemes.

Themes	Subthemes
Sounds of machines and various inanimate objects: What’s a “beep”?	Constant “beeping” as a symbol of the parents’ ceaseless worrying
	The role of “beeps” as traumatic reminders
Sounds from human sources	The sounds of medical staff
	The sounds of other hospitalized families
Unheard parents’ sounds: “we couldn’t vocalize!”	Parents’ feelings of transparency and incapability
	Parents’ perplexity towards other babies’ cry
The sound of music as balancing the NICU’s cacophony	Music as a supporting agent for parents and babies in the NICU
	Music as a transitional object for families when entering their home environments

## Supplementary Materials

The following are available online at https://www.mdpi.com/article/10.3390/children8080644/s1, Table S1A: Sounds created by Machines. Table S1B: Sounds created by Other than machines. Table S2: Summary of principal component analysis results for SON-Q (N = 386, Items = 204). Table S3: Regression models predicting the PPQ-Total Score. Questionnaire S1: Final questionnaire items (n = 77) following PCA.
