# Supplementary material for: The Soundscape of Neonatal Intensive Care: A Mixed-Methods Study of the Parents’ Experience
Source: Children (Basel). 2021 Jul 27;8(8):644. doi: 10.3390/children8080644 (PMC8391440; doi:10.3390/children8080644)
Supplement: Supplementary file 1 [file children-08-00644-s001.zip › children-1254391-supplementary.pdf]

children MDPI

The soundscape of neonatal intensive care: A mixed methods study of the parents' experience

Maria Chifa[1], Tamar Hadar[2], Nina Politimou[3], Gemma Reynolds[1] & Fabia Franco[1]
[1] Middlesex University, London (UK)
[2] Lesley University, Cambridge MA (USA),
[3] University College London (UK)

* *Correspondence: Dr Fabia Franco, Email: F.Franco@mdx.ac.uk*

SUPPLEMENTARY MATERIALS

Supplementary 1: Noises/sounds found in the Neonatal Intensive Care Unit (NICU) based on their sources: 1A) Machines, 1B) Other sources

Table S1A. Sounds created by Machines.

Description of the Equipment / Machines	The sound that it produces
**Incubator** - the box where the baby is kept.	Occasional sounds, e.g., when opening the top, when the temperature is too high, or there is some defect. M.: "The new ones we have make currently the loudest noise on the unit. It's really loud and continues until off button pressed after being unplugged" (Neonatal nurse, London, personal communication).
**Ventilator** – breathing machine for mechanical ventilation.	This can have various modes, such as high-frequency oscillation, volume guaranteed etc., depending on what works better for the child. This machine can produce a mixture of sounds: continuous vibration in case of high-frequency, continuous sound made by the humidifier and occasional alarms if the pressure drops, if the child needs resuscitation or some tubing that needs to be changed.
Bilevel Positive Airway Pressure (**BIPAP**) - provides pressurized air into airways.	Soft and rhythmic, with occasional alarms if the pressure drops or something is not working.
Continuous positive airway pressure therapy **(CPAP**) – provides less pressure than the BIPAP.	This machine can vibrate, hum, and be noisy.
Heated, humidified high-flow nasal cannula (also called HHHFNC, **high-flow** or **optiflow)** – delivers warm, moist air with or without oxygen into child lungs via a nasal cannula.	Soft, continuous sounds, with occasional alarms.
Vital signs **monitor** – for heart rate, blood pressure, breathing rate, oxygen saturation and so on.	This makes the most alarms than any other machines in NICU. It alarms if any of the previously mentioned go below or above the limit, ranging from a slow to fast rhythmic, obsessive sound.
**Infusion pumps** – delivers fluid at an established rate, in small or big quantities.	It only beeps when the infusion is about to finish, when it is finished, or if there are any blockages. The sound is loud and rhythmic.
**Tecotherm** (Freezing machine) – lowers and keeps a child`s temperature at 33` degrees	Continuous, buzzing sound.

**Feeding pump** – pushes the milk via a tube.	Soft, continuous sound.
**Portable x-ray**	Its wheels make a very low noise, and when it flashes, it makes the sound of a toy gun, just one-off.
**Portable cupboard -** with the materials necessary, e.g. for long line insertion.	Its wheels make a shaky noise.
**Portable echo** – for brain, heart scans etc.	Its wheels make a quiet and mysterious noise. Head scans particularly, one setting makes similar sounds as an ECG machine.
**Portable incubator** – it is used to transfer babies to other hospitals.	Again, only the wheels are noisy.
**Fridges –** to deposit moms` milk and medicines.	Really loud high pitch noise. Usually, it takes quite a long time to stop because if open too long, it needs to reach a certain temperature
**Apnea alarm** – to detect babies` breathing.	Beeping sound if it doesn't detect breathing for longer than 20 seconds.
**Doctors' bleeps –** special transmitters on which doctors receive various messages.	Intensive, alarming sounds until attended.
**Computers on the wheels** – used for electronic health record system.	When they run out of battery it makes a high pitch continuous beep.
**Expressing pumps –** most of the time moms express in the expressing room. However, there are cases when the pump is brought into the nursery and the expressing is done next to the incubator.	Continuous, regular sound.

Table S1B. Sounds created by Other than machines.

Sources of sound	The type of sound
**People**- nurses, doctors, relatives, including siblings, and other professionals.	Loud noise when talking.
**Bins –** they are used most of the time, such as when a nappy is changed, baby fed, treatment administered. Its bag is changed several times a day.	Banging sounds.
**Cleaning equipment**	Irregular sounds.
**Staplers**	Loud click.
**Syringes –** used for feeding and treatments (there hundreds/day opened).	When the package is opened, it makes a ripping sound.
**Sink**	Running water falling on the sink makes a calming sound.
**Chairs –** there are stools to sit for parents and big chairs, similar to sofas, for when they hold the baby or express milk.	They make a scratchy sound when moved around.
**Mobile curtain –** in case some parents require privacy or staff considers the treatment they administer cannot see others` patients relatives.	They are moved around on the wheels and make a monotonous sound.

**Plastic bags –** when parents enter the NICU, they are required to put all the things they brought to their babies (e.g., nappies, muslins, milk) in huge plastic bags and get them out of the nursery and put them in the incubator drawer.	Cracking sounds.

Table S2. Summary of principal component analysis results for SON-Q (N=386, Items=204).

Questionnaire item	F1	F2	F3	F4	F5	F6
5. 2. 1. The sound environment in NICU was too loud	.673					
5. 2. 5. The sound environment in NICU was disorientating	.638					
5. 3. 4. I would not make it quieter	.583					
5. 3. 3. I would change generic hospital sounds	.579					
5. 1. 3. NICU was encouraging/ reassuring	.568					
5. 3. 5. I would not change noise from staff	.564					
5. 2. 3. The sound environment in NICU was making me tired	.545					
5. 2. 4. The sound environment in NICU was not affecting the care I provided to my baby/s	.541					
2. 4. 3. Staff talking or laughing made me feel stressed	.509					
2. 5. 1. Morning was noisy	.506					
2. 5. 3. When a new baby was brought to the nursery it was noisy	.503					
5. 2. 2. The sound environment in NICU was not affecting my ability to communicate with the staff/my baby/s	.502					
5. 3. 1. I would not change sounds from machines	.477					
2. 4. 1. Emergency alarm made me feel stressed	.441					
2. 4. 6. The crying of other babies made me feel stressed	.439					
2. 2. 5. Stressful	.434					
2. 2.2. Reassuring	.433					
5. 1. 5. NICU was emotionally cold	.426					
5. 1. 1. NICU was aversive	.415					
2. 5. 5. Visiting time (if scheduled) was noisy	.414					
2. 2. 3. Tiring	.413					
3. 4. 8. I noticed oxygen saturation change (goes up or down)		.786				
3. 4. 6. I noticed respiration change (slower or faster)		.763				
3. 4. 7. I noticed heart rate change (slower or faster)		.747				
3. 3. 8. I noticed oxygen saturation going up or down		.695				
3. 3. 6. I noticed respiration change (slower or faster)		.644				
3. 3. 7. I noticed heart rate change (slower or faster)		.621				
3. 4. 2. I noticed smiles		.564				
3. 4. 5. I noticed movement in parts of body		.520				
3. 3. 4. I noticed crying/whimper		.478				
3. 3. 10. I noticed the baby/s to look away from the voice / cover face with hands		.443				
3. 3. 2. I noticed smiles		.443				
3. 1. 1. I noticed my baby/s to react to constant and loud noises from the machines (e.g., ventilator, CPAP)		.414				
3. 6. 3. I did sing to keep my baby/s company			.741			
3. 6. 5. I did sing to familiarise baby/s with me			.713			
3. 6. 1. I did sing to calm my baby/s			.700			
3. 6. 2. I did not sing to stimulate the baby/s, to help with their development			.699			
3. 6. 4. I did not sing to express my feelings and thoughts			.694			
3. 7. 6. I did not use recordings to cover the noises in the NICU			.681			
3. 7. 1. I used recordings to calm/soothe my baby/s			.672			
3. 7. 5. I used recordings to familiarise baby/s with me			.657			
3. 7. 3. I did use recordings to keep my baby/s company			.654			
3. 7. 2. I did not use recordings to stimulate the baby/s, to help with their development			.630			

3. 7. 4. I did not use recordings to express my feelings and thoughts			.626			
2. 8. 6. I was singing/humming			.602			
2. 11. 2. I did sing / hum to my baby/s			.601			
3. 6. 6. I did not sing to cover the noises in the NICU			.557			
3. 2. 3. I noticed my baby/s to react to my voice singing/humming			.451			
2. 11. 5. I did not make recordings to be played to my baby/s			.431			
3. 5. 1. I did speak/ talk to calm my baby/s				.556		
3. 5. 3. I did speak/ talk to keep my baby/s company				.519		
2. 11. 1. I did not talk to my baby/s				.518		
3. 5. 5. I did speak/ talk to familiarise baby/s with me				.496		
2. 8. 1. I was holding hand on her/him				.482		
2. 11. 4. I did hold hand on my baby/s (if allowed)				.447		
4. 4. 1. I have been able to discriminate pain cry				.438		
4. 5. 3. I was not talking				.420		
2. 10. 1. I was able to recognise my baby/s by his/her cry					.594	
2. 10. 3. I was able to recognise my baby/s by his/her hiccups					.519	
2. 10. 2. I was not able to recognise my baby/s by his/her whimper					.513	
2. 10. 7. I was able to recognise my baby/s by his/her laugh					.505	
2. 10. 6. I was not able to recognise my baby/s by his/her gurgling					.500	
2. 10. 5. I was able to recognise my baby/s by his/her babbling					.493	
2. 10. 4. I was not able to recognise my baby/s by his/her cooing					.442	
2. 7. 6. I have been able to discriminate tiredness cry					.436	
2. 7. 1. I have been able to discriminate pain cry					.429	
3. 3. 3. I did not notice vocalisations					.421	
2. 6. 1. My baby`s cry was loud					.404	
4. 7. 1. I found it relaxing						.722
4. 7. 3. I found it peaceful						.698
4. 7. 4. I did not find it reassuring						.670
4. 7. 5. I found it discouraging/inadequate						.585
4. 7. 7. I did not find it enjoyable						.583
4. 7. 6. I found it chaotic						.516
4. 3. 1. I thought that baby/s cried more often						.467
4. 3. 5. I thought the baby's cry was easier to calm						.467
**Eigenvalues**	7.924	5.374	4.065	2.632	2.557	2.123
**% of variance**	10.208	10.540	11.452	9.431	9.146	7.655
**α**	.867	.900	.913	.754	.810	.815

Key: items number represents, in order respectively, the part of the questionnaire with Likert-scale items (2 = You and sound: Going to NICU; 3 = Your baby and sound in NICU; 4 = At home after NICU; 5 = About NICU in general,), the question (overall n = 32), and the individual item (presented with a 5-point Likert scale) – e.g., the first item in the table is from part 5 (About NICU in general), question 2 (How would you describe the sound environment in NICU? Please rate the following statements.), item 1.

Questionnaire S1: Final questionnaire items (*n*= 77) following PCA.

Note: original item number is presented in brackets, e.g., [5.2.1], see Table S2.

1. How would you describe the sound environment in NICU? Please rate the following statements.
   - *The sound environment in NICU was too loud [5.2.1]*
   - *The sound environment in NICU was disorientating [5. 2.].]*
   - *The sound environment in NICU was making me tired [5. 2. 3.]*
   - *The sound environment in NICU was not affecting the care I provided to my baby/s [5. 2. 4.]*
   - *The sound environment in NICU was not affecting my ability to communicate with the staff/my baby/s [5. 2. 2.]*

2. If you had the possibility, how would you change the sound environment in NICU? Please rate the following statements.
   - *I would not make it quieter [5. 3. 4.]*

- *I would change generic hospital sounds [5. 3. 3]*
- *I would not change noise from staff [5. 3. 5]*
- *I would not change sounds from machines [5. 3. 1.]*

3. How would you describe the NICU environment in general? Please rate the following statements.
- *NICU was encouraging/ reassuring* [5. 1. 3.]
- *NICU was emotionally cold [5. 1. 5.]*
- *NICU was aversive [5. 1. 1.]*

4. How stressed/anxious did the following sounds make you feel?
- *Staff talking or laughing made me feel stressed [2. 4. 3.]*
- *Emergency alarm made me feel stressed [2. 4. 1.]*
- *The crying of other babies made me feel stressed* [2. 4. 6.]

5. If your baby/s was premature or sick and went to NICU, how would you describe your general experience of the sounds there?
- *Stressful [2. 2. 5.]*
- *Reassuring [2. 2.2.]*
- *Tiring [2. 2. 3.]*

6. How noisy was NICU in the situations below?
- *Morning was noisy [2. 5. 1.]*
- *When a new baby was brought to the nursery it was noisy [2. 5. 3.]*
- *Visiting time (if scheduled) was noisy [2. 5. 5.]*

7. Please score the different types of reactions that you noticed in your baby/s towards <u>the noises in the NICU</u> (e.g. from machines, opening incubator, doors, tap water etc.)? Please, score the below reactions.
- *I noticed oxygen saturation change (goes up or down) [3. 4. 8.]*
- *I noticed respiration change (slower or faster)* [3. 4. 6.]
- *3. I noticed heart rate change (slower or faster) [3. 4. 7.]*
- *I noticed smiles [3. 4. 2.]*
- *I noticed movement in parts of body [3. 4. 5.]*

8. Please describe the different types of reactions that you noticed in your baby/s when s/he <u>listened to voices</u> speaking or singing to her/him in the NICU?
- *I noticed oxygen saturation going up or down [3. 3. 8.]*
- *I noticed respiration change (slower or faster) [3. 3. 6.]*
- *I noticed heart rate change (slower or faster)* [3. 3. 7.]
- *I noticed crying/whimper [3. 3. 4.]*
- *I noticed the baby/s to look away from the voice / cover face with hands [3. 3. 10.]*
- *I noticed smiles [3. 3. 2.]*
- *I did not notice vocalisations [3. 3. 3.]*

9. Please, indicate if your baby/s reacted to the following <u>noises</u> while in NICU.
- *I noticed my baby/s to react to constant and loud noises from the machines (e.g., ventilator, CPAP) [3. 1. 1.]*

10. Did you <u>sing</u> to your baby/s while in the NICU? Please, rate the options below to indicate the reasons you might have had to sing to your baby/s.
- *I did sing to keep my baby/s company [3. 6. 3.]*
- *I did sing to familiarise baby/s with me [3. 6. 5.]*
- *I did sing to calm my baby/s [3. 6. 1.]*
- *I did not sing to stimulate the baby/s, to help with their development [3. 6. 2.]*
- *I did not sing to express my feelings and thoughts [3. 6. 4.]*

- *I did not sing to cover the noises in the NICU [3. 6. 6.]*

11. Did you use <u>recordings</u> of your voice or music for your baby/s while in the NICU? Please, rate the options below. If you did not use recordings, please skip and go to the following question.
    - *I did not use recordings to cover the noises in the NICU [3. 7. 6.]*
    - *I used recordings to calm/soothe my baby/s [3. 7. 1.]*
    - *I used recordings to familiarise baby/s with me [3. 7. 5.]*
    - *I did use recordings to keep my baby/s company [3. 7. 3.]*
    - *I did not use recordings to stimulate the baby/s, to help with their development [3. 7. 2.]*
    - *I did not use recordings to express my feelings and thoughts [3. 7. 4.]*

12. Did you do the following to comfort/calm your baby/s in NICU?
    - *I was singing/humming [2. 8. 6.]*
    - *I was holding hand on her/him [2. 8. 1.]*

13. How did you communicate with your baby/s at the NICU?
    - *I did sing / hum to my baby/s [2.11. 2.]*
    - *I did not make recordings to be played to my baby/s [2.11. 5.]*
    - *I did not talk to my baby/s [2. 11. 1.]*
    - *I did hold hand on my baby/s (if allowed) [2. 11. 4.]*

14. Please indicate how your baby/s reacted to the following sounds from <u>people</u> in the NICU.
    - *I noticed my baby/s to react to my voice singing/humming [3. 2. 3.]*

15. Did you or any other person <u>speak</u> to your baby/s while in the NICU? Please, rate the options below to indicate the reasons you might have had to speak to your baby/s.
    - *I did speak/ talk to calm my baby/s [3. 5. 1.]*
    - *I did speak/ talk to keep my baby/s company [3. 5. 3.]*
    - *I did speak/ talk to familiarise baby/s with me [3. 5. 5.]*

16. At home, have you been able to discriminate different types within your baby`s crying?
    - *I have been able to discriminate pain cry [4. 4. 1.]*

17. How did you comfort/calm your baby/s when crying at home?
    - *I was not talking [4. 5. 3.]*

18. When approaching the nursery your baby/s was in, did any of the following sounds make recognise her/him?
    - *I was able to recognise my baby/s by his/her cry [2. 10. 1.]*
    - *I was able to recognise my baby/s by his/her hiccups [2. 10. 3.]*
    - *I was not able to recognise my baby/s by his/her whimper [2. 10. 2.]*
    - *I was able to recognise my baby/s by his/her laugh [2. 10. 7.]*
    - *I was not able to recognise my baby/s by his/her gurgling [2. 10. 6.]*
    - *I was able to recognise my baby/s by his/her babbling [2. 10. 5.]*
    - *I was not able to recognise my baby/s by his/her cooing [2. 10. 4.]*

19. Have you been able to discriminate different types of cry within your baby`s crying in NICU?
    - *I have been able to discriminate tiredness cry [2. 7. 6.]*
    - *I have been able to discriminate pain cry [2. 7. 1.]*

20. If your baby/s was able to cry, how was her/his crying in NICU?
    - *My baby`s cry was loud [2. 6. 1.]*

21. Compared with the NICU, how did you find your home environment when home?

- *I found it relaxing [4. 7. 1.]*
- *I found it peaceful [4. 7. 3.]*
- *I did not find it reassuring [4. 7. 4.]*
- *I found it discouraging/inadequate [4. 7. 5.]*
- *I did not find it enjoyable [4. 7. 7.]*
- *I found it chaotic [4. 7. 6.]*

22. Did you think that your baby's crying changed once at home? Please rate the following options.
- *I thought that baby/s cried more often [4. 3. 1.]*
- *I thought the baby's cry was easier to calm* [4. 3. 5.]

N.B. Negative items were reversed for scoring.

Table S3. Regression models predicting the PPQ -Total Score.

Model		B	β	t	Sig.	F change	Sig. *F* change
1						24.51	0.000
	Parental Age	−0.24	−0.099	−2.19	0.029		
	NICU soundscape	0.58	0.46	10.27	0.000		
	Infant_Reactions	−0.06	-0.05	−1.09	0.277		
	Singing – Recording	0.01	0.01	0.21	0.831		
	Bonding with Baby	0.48	0.11	2.42	0.016		
	Parenting Confidence	−0.05	−0.03	−0.63	0.525		
	Home Environment	−0.60	−0.28	−6.45	0.000		
2						0.04	0.831
	Parental Age	−0.24	−0.09	−2.19	0.029		
	NICU soundscape	0.58	0.46	10.30	0.000		
	Infant Reactions	−0.06	−0.05	−1.07	0.285		
	Bonding with Baby	0.49	0.11	2.56	0.011		
	Parenting Confidence	−0.04	−0.02	−0.60	0.544		
	Home Environment	−0.60	−0.28	−6.48	0.000		
3						0.36	0.544
	Parental Age	−0.22	-0.09	−2.11	0.035		
	NICU soundscape	0.59	0.47	10.50	0.000		
	Infant_Reactions	−0.07	−0.06	−1.33	0.184		
	Bonding with Baby	0.48	0.11	2.51	0.012		
	Home Environment	−0.61	−0.29	−6.61	0.000		
4						1.76	0.184
	Parental Age	−0.20	−0.08	−1.93	0.054		
	NICU soundscape	0.57	0.45	10.60	0.000		
	Bonding with Baby	0.45	0.10	2.35	0.019		
	Home Environment	−0.60	−0.28	−6.54	0.000		

The independent variables gradually eliminated are: Singing – Recordings", "Parenting Confidence" and "Infant Reactions"
